# Different effects of anesthetic isoflurane on caspase-3 activation and cytosol cytochrome c levels between mice neural progenitor cells and neurons

**DOI:** 10.3389/fncel.2014.00014

**Published:** 2014-01-30

**Authors:** Yiying Zhang, Chuxiong Pan, Xu Wu, Yuanlin Dong, Deborah J. Culley, Gregory Crosby, Tianzuo Li, Zhongcong Xie

**Affiliations:** ^1^Geriatric Anesthesia Research Unit, Department of Anesthesia, Critical Care and Pain Medicine, Massachusetts General Hospital and Harvard Medical SchoolCharlestown, MA, USA; ^2^Department of Anesthesia, Beijing Tongren Hospital, Capital Medical UniversityBeijing, China; ^3^Department of Forensic Pathology, Faculty of Forensic Medicine, China Medical University, Shenyang, China; ^4^Department of Anesthesia, Brigham and Women’s Hospital and Harvard Medical SchoolBoston, MA, USA

**Keywords:** Alzheimer’s disease, anesthesia, isoflurane, caspase-3, cytochrome c, neural progenitor cells, neurons

## Abstract

Commonly used anesthetic isoflurane has been reported to promote Alzheimer’s disease (AD) neuropathogenesis by inducing caspase-3 activation. However, the up-stream mechanisms of isoflurane’s effects remain largely to be determined. Specifically, there is a lack of a good model/system to elucidate the underlying mechanism of the isoflurane-induced caspase-3 activation. We therefore set out to assess and compare the effects of isoflurane on caspase-3 activation in neural progenitor cells (NPCs) and in primary neurons from wild-type (WT) and AD transgenic (Tg) mice. The NPCs and neurons were obtained, cultured and then treated with either 2% isoflurane or under control condition for 6 h. The NPCs or neurons were harvested at the end of the treatment and were subjected to Western blot analysis. Here we showed for the first time that the isoflurane treatment induced caspase-3 activation in neurons, but not in NPCs, from either WT or AD Tg mice. Consistently, the isoflurane treatment increased cytosol levels of cytochrome c, a potential up-stream mechanism of isoflurane-induced caspase-3 activation in the mice neurons, but not NPCs. Finally, the isoflurane treatment induced a greater casapse-3 activation in the neurons, but not the NPCs, from AD Tg mice as compared to the WT mice. These data demonstrated that investigation and comparison of isoflurane’s effects between mice NPCs and neurons would serve as a model/system to determine the underlying mechanism by which isoflurane induces caspase-3 activation. These findings would promote more research to investigate the effects of anesthetics on AD neuropathogenesis and the underlying mechanisms.

## Introduction

Alzheimer’s disease (AD) is one of the most common forms of dementia. Early studies showed that the age of onset of AD was inversely related to the cumulative exposure to general anesthesia before the age of 50 (Bohnen et al., 1994b; Muravchick and Smith, 1995). Lee et al. illustrated that patients having coronary artery bypass graft surgery under general anesthesia were at increased risk for the emergence of AD compared to those having percutaneous transluminal coronary angioplasty under local anesthesia (Lee et al., 2005). However, other studies showed that there was no association between anesthesia/surgery and AD (Bohnen et al., 1994a; Knopman et al., 2005; Avidan et al., 2009; Sprung et al., 2013). Recently, Chen et al. analyzed the claims data of 1 million insured residents covered by Taiwan’s universal health insurance from 2005 to 2009 and found that previous exposure to surgery under general anesthesia might be associated with an increased risk of AD, particularly in patients who had undergone repeated exposure to general anesthesia (Chen et al., 2013a). Moreover, in a prospective and randomized study of stable mild cognitive impairment patients, Liu et al. found that patients who had sevoflurane anesthesia for surgery showed a significantly faster progression of cognitive function decline as compared to the patients who had epidural anesthesia, propofol anesthesia, or no anesthesia (Liu et al., 2013). It has been shown that there is a greater gray matter loss and decline of cognitive function following anesthesia and surgery as compared to an age-matched control cohort (Kline et al., 2012). Finally, a retrospective study, including 24,901 patients in the anesthesia/surgery group and 110,972 participants in the control group, has demonstrated that patients who undergo anesthesia and surgery may be at increased risk for dementia (hazard ratio = 1.99; Chen et al., 2013b). Moreover, anesthesia and surgery have been reported to cause cognitive dysfunction, which AD patients are susceptible to develop (Moller et al., 1998; reviewed in Terrando et al., 2011; Eckenhoff and Planel, 2013). Collectively, it is important to study whether anesthetics can promote AD neuropathogenesis and investigate the underlying mechanisms.

A recent study suggests that caspase-3 activation even without apoptosis can contribute to AD neuropathogenesis (Burguillos et al., 2011). The commonly used inhalation anesthetic isoflurane has been shown to promote AD neuropathogenesis, including induction of caspase-3 activation and apoptosis (Eckenhoff et al., 2004; Loop et al., 2005; Xie et al., 2006a, 2007, 2008; Wei et al., 2008; Zhang et al., 2010, 2012). However, the underlying mechanism by which isoflurane induces caspase-3 activation still remains largely to be determined. Moreover, there is a lack of a good model/system that can be used to determine the underlying mechanism(s) of the isoflurane-induced caspase-3 activation. Neural progenitor cells (NPCs) may have resistance to caspase-3 activation and apoptosis (Peng et al., 2005). We therefore have hypothesized that isoflurane induces caspase-3 activation in neurons but not in NPCs, and such a difference can be used to establish a model/system to investigate the underlying mechanism, e.g., cytosol cytochrome c levels, by which isoflurane induces caspase-3 activation. We have utilized NPCs and primary neurons from wild-type (WT) mice and AD transgenic (Tg) mice [B6.Cg-Tg (APPswe, PSEN1dE9) 85Dbo/J]. The AD Tg mice have the same genetic background as the WT mice (C57BL/6J) but with two mutant genes linked to familial AD: *APP* and *PSEN1* (Garcia-Alloza et al., 2006; Xiong et al., 2011). It has been reported that another anesthetic, sevoflurane, induces a greater caspase-3 activation in the brain tissues of the young (6 day-old) AD Tg mice than that of WT mice (Lu et al., 2010). Therefore, we used the AD Tg mice to determine whether isoflurane could induce a greater caspase-3 activation in the NPCs or neurons of the AD Tg mice than those of the WT mice.

## Materials and Methods

### Mice in the studies

The animal protocol was approved by the Standing Committee on Animals at Massachusetts General Hospital, Boston, Massachusetts. WT mice (C57BL/6J, The Jackson Lab, Bar Harbor, ME) and AD Tg mice [B6.Cg-Tg (APPswe, PSEN1dE9) 85Dbo/J (The Jackson Laboratory)] were used in the study. WT and AD Tg mice were distinguished by genotyping.

### “Complete” proliferation media to culture Neural progenitor cells (NPCs)

Fifty ml NeuroCult™ neural stem cell proliferation supplements (Stem Cell Technologies Inc., Vancouver, B.C., Canada) and 200 μg human epidermal growth factor (hEGF) (Stem Cell Technologies Inc.) were added into 450 ml NeuroCult™ neural stem cell basal media (Stem Cell Technologies Inc.) to a final concentration of 400 ng/ml of hEGF, 100 units/ml penicillin, and 100 μg/ml streptomycin in the culture media.

### Harvest of mice Neural progenitor cells (NPCs)

Harvest of mice NPCs was performed using our previous study methods, with modifications (Zhang et al., 2013). Specifically, 18 or 19 day-gestation stage mice were euthanized with carbon dioxide. Embryos were extracted using cesarean section, and then decapitated in a 100 mm dish of phosphate-buffered saline (PBS). The heads were placed in a 100 mm dish and the hippocampi were isolated. The hippocampi were put into a 15 ml tube with 2.5 ml of “complete” proliferation media, triturated 10–15 times using a 1000 μl plastic pipette tip, and filtered through a 70 μm cell strainer to obtain the suspended single NPCs. The NPCs were then suspended in 5 ml of “complete” proliferation media and a cell count was taken with a hemocytometer. One million cells were plated in each well of a six-well plate containing 2.5 ml of “complete” proliferation media. NPCs were passaged twice (every 7 days) before use. For passaging the cells, media and cells were removed and placed into a 50 ml centrifuge tube to remove adherent NPCs and dead cells. Only non-adherent proliferating NPCs were collected. The collected proliferating NPCs were centrifuged at 3000 rapid per minute (rpm) for 5 min. The medium was removed and the cells were re-suspended in 3 ml of “complete” proliferation media. The cells were then triturated 10–15 times using a 1000 μl plastic pipette tip and then plated at one million cells in 2.5 ml of “complete” proliferation media in each well of a six-well plate.

### Harvest of mice primary neurons

The mice primary neurons were harvested using the method described in our previous studies with modifications (Zhen et al., 2009; Zhang et al., 2010). Specifically, 15 day-gestation stage mice were euthanized with carbon dioxide. Embryos were extracted using cesarean section, and then decapitated in a 100 mm dish of PBS. The heads were placed in a 100 mm dish and the hippocampi were isolated. We collected the cortex, removed meninges, and placed the tissues into 100-mm dish of PBS. The neurons were dissociated by trypsinization and trituration then suspended in 5 ml of serum/neurobasal medium and a cell count was taken with a hemocytometer. The amount of 0.3 million cells were resuspended and plated into six-well plates containing 1.5 ml of serum/neurobasal medium with a confluent rate of 50%. After 1 h, the medium was changed to 1.5 ml per well serum-free B27/neurobasal medium. The neurons were exposed to the isoflurane treatment 6–8 days after the harvest.

### Treatments for Neural progenitor cells (NPCs) and neurons

The isoflurane treatment was performed as described in our previous studies (Xie et al., 2006a, 2007, 2008; Zhang et al., 2010, 2012). Specifically, isoflurane was delivered from an anesthesia machine to a sealed plastic box in a 37^˚^C incubator. The box containing six-well plates seeded with one million NPCs or 0.25–0.3 million neurons in 1.5 ml cell culture media. A Date infrared gas analyzer (Ohmeda, Tewksbury, MA) was used to continuously monitor the delivered concentrations of carbon dioxide, oxygen, and isoflurane. The mice NPCs or neurons were treated with 2% isoflurane plus 21% O_2_ and 5% CO_2_ or under control condition for 6 h. The control condition was 21% O_2_ plus 5% CO_2_.

### Immunofluorescence staining

The NPCs were washed with 3% bovine serum albumin (BSA) in PBS twice, then the NPCs were permeabilized with 0.2% Triton X-100 in PBS at 4^˚^C for 10 min, and blocked with 3% BSA at room temperature for 1 h. NPCs were further incubated with nestin antibody (1:200, Chemicon, Temecula, CA) overnight at 4^˚^C, followed by staining with Alexa Fluor® 594 goat anti-mouse IgG (Invitrogen, Carlsbad, CA) for 1 h at room temperature and away from light. Finally, the cells were incubated with 4’, 6-Diamidino-2-Phenylindole, Dihydrochloride (DAPI) in a humidified dark chamber for 10 min, and then were analyzed in mounting medium under a fluorescence microscope.

### Cellular Fraction

The cytochrome c levels in the cytosol were detected. We used mitochondrial isolation kit (Pierce, Iselin, NJ) to isolate mitochondria from cytosol, which allowed us to exclusively detect cytochrome c levels in cytosol. Specifically, the harvested neurons or NPCs after the treatment with isoflurane or control condition were placed in a 1.5 ml microcentrifuge tube, and each tube was centrifuged at 850 g for 2 min. The supernatant was removed and discarded from each tube. Then we added 400 μl of the mitochondrial isolation reagent A into each tube. After 5 s of vortex, the tubes were incubated on ice for 2 min. Then we added 0.5 ml of the mitochondrial isolation reagent B into each tube, and then they were put in a vortex at a maximum speed for 5 s. We incubated the tubes on ice for 5 min before adding 400 μl of the mitochondrial isolation reagent C into each tube, and they were centrifuged at 700 g for 10 min at 4^˚^C. We then transferred the supernatant to a new 1.5 ml tube and centrifuged each of these tubes at 3000 g for 15 min. Finally, we collected the supernatant, which contained the cytosol fraction, and discarded the pellet, which contained the isolated mitochondria. We used a Spin-X UF 500 concentrator (Corning, Lowell, MA), which could enhance the concentration of cytochrome c levels in cytosol for the Western blot analysis.

### Cell lysis and protein amount quantification

The pellets of the harvested neurons and NPCs were detergent-extracted on ice using an immunoprecipitation buffer (10 mM Tris-HCl, pH 7.4, 150 mM NaCl, 2 mM EDTA, 0.5% Nonidet P-40) plus protease inhibitors (1 μg/ml aprotinin, 1 μg/ml leupeptin, 1 μg/ml pepstatin A). The lysates were collected, centrifuged at 13,000 rpm for 15 min, and quantified for total protein amount by a bicinchoninic acid protein assay kit (Pierce).

### Western blots analysis

The harvested NPCs and neurons were subjected to Western blot analyses as described in our previous studies (Zhang et al., 2010, 2012). Specifically, a caspase-3 antibody (1:1000 dilution; Cell Signaling Technology, Danvers, MA) was used to recognize full-length caspase-3 (35–40 kDa) and caspase-3 fragment (17–20 kDa) resulting from cleavage at aspartate position 175. Rabbit polyclonal cytochrome c antibody (1:1000 dilution; Cell Signaling Technology) was used to recognize cytochrome c (14 kDa). Antibody anti-β-Actin (1:10,000, Sigma, St. Louis, MO) was used to detect β-Actin (42 kDa). Each band in the Western blot represented an independent experiment. The results were averaged from three to five independent experiments. The intensity of signals was analyzed using the National Institute of Health image program. We quantified the Western blots in two steps. First, we used β-Actin levels to normalize protein levels (e.g., determining the ratio of caspase-3 fragment to β-Actin amount) and to control for loading differences in the total protein amount. Second, we presented protein level changes in neurons or NPCs having anesthesia as a percentage of those in the control group. 100% of protein level changes refer to control levels for the purpose of comparison to experimental conditions.

### Statistics

Given the presence of background caspase-3 activation in cells, we did not use absolute values to describe these changes. Instead, the caspase-3 activation was presented as a percentage of that of the control group. Data were expressed as mean ± standard deviation (SD). The number of samples varied from three to five. Student *t*-test was used to analyze the difference between control condition and isoflurane treatment on caspase-3 activation and levels of cytosol cytochrome c. P-values less than 0.05 was considered statistically significant. Prism 6 software (La Jolla, CA) was used to analyze the data.

## Results

**Figure 1:**
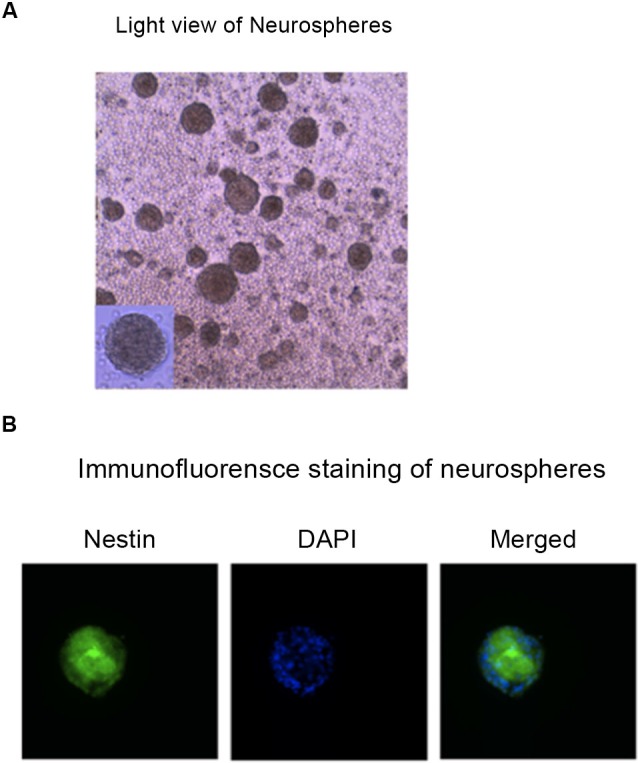
**Identification of the mouse NPCs.** Photomicrographs of hippocampus NPCs grown in the “complete” proliferation culture media. **(A)** Phase contrast photograph taken before fixation of a floating neurosphere grown in a six-well plate. **(B)** Immunofluorescence image of a neurosphere that was fixed and expressed nestin (the marker of NPCs, green), DAPI (the marker of nuclei, blue), and merged image of a neurosphere plated on FluoroDish. NPCs, neural progenitor cells; DAPI, 4’, 6-Diamidino-2-Phenylindole, Dihydrochloride.

We aimed to establish a model/system in mice NPCs and mice neurons, which could be used to determine the up-stream mechanisms of isoflurane-induced caspase-3 activation. First, we identified the mice NPCs by using immunofluorescence staining of neurospheres with nestin (Figure 1).

### Isoflurane induced caspase-3 activation in mice neurons but not in mice Neural progenitor cells (NPCs)

**Figure 2:**
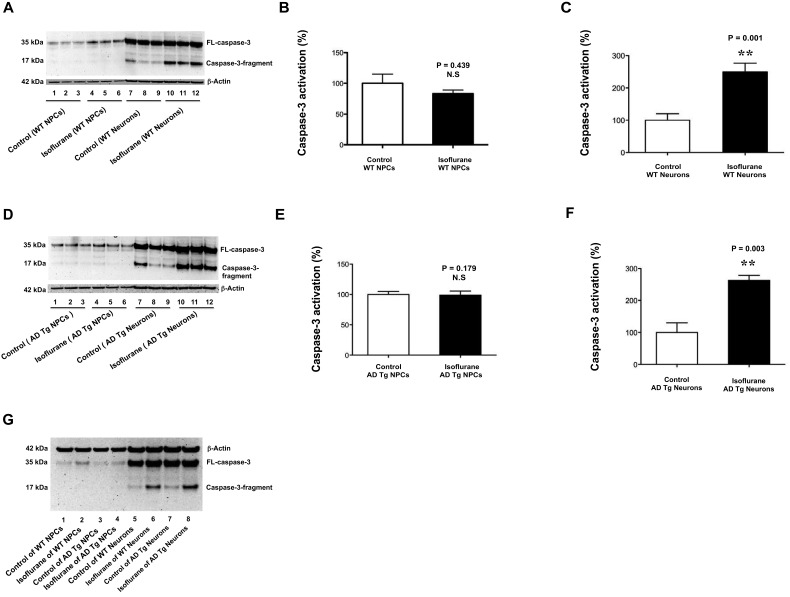
**Isoflurane induces caspase-3 activation in mice neurons but not in mice NPCs. (A)** There is no visible difference in the caspase-3 activation (defined as the ratio of caspase-3 fragment to full-length caspase-3) between the control condition (lanes 1–3) and the isoflurane treatment (lanes 4–6) in the WT mice NPCs. There is a visible increase in the caspase-3 fragment following the isoflurane treatment (lanes 10–12) as compared to that following control condition (lanes 7–9) in the WT mice neurons. **(B)** The quantification of the Western blot shows that the isoflurane treatment (black bar) does not induce caspase-3 activation as compared to control condition (white bar) in the WT mice NPCs. **(C)** The quantification of the Western blot shows that the isoflurane treatment (black bar) induces caspase-3 activation as compared to the control condition (white bar) in WT mice neurons. **(D)** There is no visible difference in the caspase-3 activation (defined as the ratio of caspase-3 fragment to full-length caspase-3) between the control condition (lanes 1–3) and the isoflurane treatment (lanes 4–6) in the AD Tg mice NPCs. There is a visible increase in the caspase-3 fragment following the isoflurane treatment (lanes 10–12) as compared to that following control condition (lanes 7–9) in the AD Tg mice neurons. **(E)** The quantification of the Western blot shows that the isoflurane treatment (black bar) does not induce caspase-3 activation as compared to control condition (white bar) in the AD Tg mice NPCs. **(F)** The quantification of the Western blot shows that the isoflurane treatment (black bar) induces caspase-3 activation as compared to the control condition (white bar) in AD Tg mice neurons. **(G)** There is a higher level of caspae-3 fragment following the isoflurane treatment (lane 6 or 8) than that following control condition (lane 5 or 7) in the WT or AD Tg mice neurons. The caspase-3 fragment level following the isoflurane treatment in the AD Tg mice neurons (lane 8) is greater than that in the WT mice neurons (lane 6). There is no visible difference in the levels of caspase-3 fragment between control condition and isoflurane treatment in the WT mice NPCs or AD Tg mice NPCs (lanes 1–4). NPCs, neural progenitor cells; AD, Alzheimer’s disease; Tg, transgenic; WT, wild type; full-length, FL.

After identification of NPCs, we then determined and compared the effects of isoflurane on caspase-3 activation in NPCs and neurons from WT mice. Immunoblotting of caspase-3 showed that the isoflurane treatment (2% isoflurane for 6 h, lanes 4–6) did not induce caspase-3 activation, as evidenced by the increased ratio of caspase-3 fragment to full-length (FL) caspase-3, compared with the control condition (lanes 1–3) in the NPCs from WT mice (Figure 2A). However, the same isoflurane treatment (lanes 10–12) induced caspase-3 activation as compared to the control condition (lanes 7–9) in the neurons from the WT mice (Figure 2A). The quantification of the Western blot showed that the isoflurane treatment (black bar) did not induce caspase-3 activation as compared to control condition (white bar) in WT mice NPCs: 83% versus 100%, *P* = 0.439, N.S. (Figure 2B). In the WT mice neurons, the quantification of the Western blot showed that the isoflurane treatment (black bar) induced caspase-3 activation as compared to control condition (white bar): 250% versus 100%, ** *P* = 0.001 (Figure 2C). These data suggested that whereas isoflurane was able to induce caspase-3 activation in WT mice neurons, the isoflurane treatment was not able to induce caspase-3 activation in WT mice NPCs.

Our previous studies showed that sevoflurane, another commonly used inhalation anesthetic, induced a greater caspase-3 activation in the brain tissues of 6 day-old AD Tg mice than that of 6 day-old WT mice (Lu et al., 2010). We therefore asked whether isoflurane could induce caspase-3 activation in AD Tg mice NPCs. Immunoblotting of caspase-3 showed that isoflurane treatment (2% isoflurane for 6 h, lanes 4–6) did not induce caspase-3 activation as compared to control condition (lanes 1–3) in the AD Tg mice NPCs (Figure 2D). In the AD Tg mice neurons, however, the isoflurane treatment (lanes 10–12) induced caspase-3 activation as compared to the control condition (lanes 7–9) (Figure 2D). The quantification of the Western blot showed that the isoflurane treatment (black bar) did not induce caspase-3 activation as compared to the control condition (white bar) in the AD Tg mice NPCs: 96% versus 100%, *P* = 0.179, N.S. (Figure 2E). In the AD Tg mice neurons, however, the quantification of the Western blot showed that the isoflurane treatment (black bar) induced caspase-3 activation as compared to control condition (white bar): 270% versus 100%, ** *P* = 0.003 (Figure 2F). These data further suggested that isoflurane had different effects on caspase-3 activation in the NPCs and neurons from both WT and AD Tg mice.

Our previous studies have shown that anesthetic sevoflurane induces a greater caspase-3 activation in the brain tissues of AD Tg mice (Lu et al., 2010). We therefore asked whether isoflurane could also induce a greater caspase-3 activation in the NPCs and neurons of AD Tg mice than those of WT mice by using a side-by-side comparison of isoflurane’s effects on caspase-3 activation between NPCs and neurons of WT or AD Tg mice. The caspase-3 immunoblotting showed that the isoflurane treatment (lane 6, WT mice neurons, lane 8, AD Tg mice neurons) induced caspase-3 activation as compared to control condition (lane 5, WT mice neurons, lane 7 AD Tg mice neurons) (Figure 2G). Moreover, the isoflurane treatment induced a greater caspase-3 activation in AD Tg neurons than that in WT neurons: lane 8 versus lane 6 (Figure 2G). However, the isoflurane treatment did not cause a difference in caspase-3 activation between WT mice NPCs and AD Tg mice NPCs (Figure 2G). These data suggested that isoflurane induced a greater caspase-3 activation in AD Tg mice neurons than WT mice neurons. However, even in the NPCs from the AD Tg mice, the isoflurane treatment did not induce caspase-3 activation.

### Isoflurane increased cytosol cytochrome c levels in mice neurons but not in mice Neural progenitor cells (NPCs)

**Figure 3:**
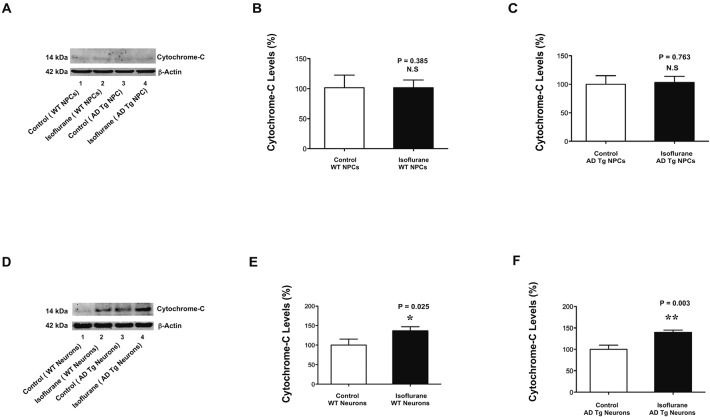
**Isoflurane increases cytochrome c levels in mice neurons but not in mice NPCs.(A)** There is no visible difference in the cytosol cytochrome c levels between the control condition (lane 1) and the isoflurane treatment (lane 2) in the WT mice NPCs. There is no visible difference in the cytochrome c levels between the control condition (lane 3) and the isoflurane treatment (lane 4) in the AD Tg mice NPCs. **(B)** The quantification of the Western blot shows that the isoflurane treatment (black bar) does not increase cytosol cytochrome c level as compared to control condition (white bar) in the WT mice NPCs. **(C)** The quantification of the Western blot shows that the isoflurane treatment (black bar) does not increase cytosol cytochrome c levels as compared to the control condition (white bar) in AD Tg mice NPCs. **(D)** There is a visible increase in the cytosol cytochrome c levels following the treatment of isoflurane (lane 2) as compared to that following the control condition (lane 1) in the WT mice neurons. There is a visible increase in the cytosol cytochrome c levels following the treatment of isoflurane (lane 4) as compared to that following the control condition (lane 3) in the AD Tg mice neurons. **(E)** The quantification of the Western blot shows that the isoflurane treatment (black bar) increases cytosol cytochrome c levels as compared to control condition (white bar) in the WT mice neurons. **(F)** The quantification of the Western blot shows that the isoflurane treatment (black bar) increases cytosol cytochrome c levels as compared to the control condition (white bar) in the AD Tg mice neurons. NPCs, neural progenitor cells; AD, Alzheimer’s disease; Tg, transgenic; WT, wild-type.

The findings that isoflurane induced caspase-3 activation in mice neurons but not in mice NPCs suggested that the comparison of isoflurane’s effects between mice neurons and mice NPCs could be used to determine the underlying mechanisms of isoflurane’s neurotoxicity. Our previous studies have shown that treatment with 2% isoflurane for 6 h induces caspase-3 activation and increases cytosol levels of cytochrome c in H4 human neuroglioma cells (Zhang et al., 2010). However, whether the isoflurane-induced caspase-3 activation is associated with the isoflurane-induced elevation of cytosol cytochrome c is unknown. Therefore, we investigated and compared the effects of isoflurane on cytosol cytochrome c levels between mice NPCs and mice neurons. The immunoblotting of cytochrome c showed that there was no visible difference in the cytosol levels of cytochrome c between control condition (lane 1 or 3 of Figure 3A) and isoflurane treatment (lane 2 or 4, Figure 3A) in either WT mice NPCs (lanes 1 and 2) or AD Tg mice NPCs (lanes 3 and 4) (Figure 3A). The quantification of the Western blot showed that the isoflurane treatment did not increase the cytosol levels of cytochrome c in WT mice NPCs (Figure 3B) and in AD Tg mice NPCs (Figure 3C). In the WT mice neurons, however, the immunoblotting of cytochrome c showed that the isoflurane treatment (lane 2, Figure 3D) induced a visible increase in the band of cytosol cytochrome c as compared to the control condition (lane 1, Figure 3D). Moreover, in the AD Tg mice neurons, the cytochrome c immunoblotting showed that the isoflurane treatment (lane 4, Figure 3D) induced a visible increase in the band of cytosol cytochrome c as compared to control condition (lane 3, Figure 3D). The quantification of the Western blot showed that the isoflurane treatment (black bar) increased the cytosol levels of cytochrome c as compared to the control condition (white bar) in WT mice neurons (Figure 3E): 140% versus 100%, * *P* = 0.025; and in AD Tg mice neurons (Figure 3F): 143% versus 100%, ** *P* = 0.003. These data suggested that isoflurane only increased cytosol cytochrome c levels in mice neurons but not in mice NPCs.

## Discussion

The objective of the current study was to establish a model/system to determine the underlying mechanism by which isoflurane, a commonly used inhalation anesthetic, induces caspase-3 activation. We found that the treatment with 2% isoflurane for 6 h was able to induce caspase-3 activation in WT mice neurons, but not in WT mice NPCs (Figure 2). These results suggested that the difference in caspase-3 activation following the isoflurane treatment between mice NPCs and neurons could serve as a model/system to determine the up-stream mechanisms of the isoflurane-induced caspase-3 activation.

Our previous studies have shown that isoflurane can induce caspase-3 activation and elevate the cytosol cytochrome c levels in H4 human neuroglioma cells (Zhang et al., 2010). However, whether the isoflurane-induced caspase-3 activation is associated with the isoflurane-induced elevation of cytosol cytochrome c levels remains unknown, owing to the lack of a model/system to perform such studies. In the current experiments, we found that the isoflurane treatment induced neither caspase-3 activation nor elevation of cytosol cytochrome c levels in WT mice NPCs (Figures 2 and 3). On the other hand, the isoflurane treatment induced caspase-3 activation as well as elevation of cytosol cytochrome c levels in WT mice neurons (Figures 2 and 3). Taken together, these data suggested that the isoflurane-induced caspase-3 activation was associated with the isoflurane-induced elevation of cytosol cytochrome c levels.

More importantly, these findings demonstrated that we have established a model/system in NPCs and neurons, and we would be able to use the established model/system to further determine the up-stream mechanisms of the isoflurane-induced caspase-3 activation in the future. Our previous studies suggested that isoflurane might induce mitochondrial dysfunction, including accumulation of reactive oxygen species and opening of mitochondrial permeability transition pore, leading to caspase-3 activation (Zhang et al., 2012). Thus, our future studies would further test this hypothesis by determining and comparing the effects of isoflurane on the accumulation of reactive oxygen species and opening of the mitochondrial permeability transition pore between mice NPCs and mice neurons.

Mutations of *APP* and *PSEN1* gene may increase the vulnerability to anesthetic (Lu et al., 2010). Consistently, we found that isoflurane induced a greater caspase-3 activation in the neurons harvested from the AD Tg mice with mutation of AD associated genes *APP* and *PSEN1* than that in neurons harvested from WT mice in the current studies (Figure 2G). We also found that the isoflurane treatment did not induce caspase-3 activation and elevation of cytosol cytochrome c levels even in the NPCs from AD Tg mice (Figures 2D; and 3A). Collectively, these data suggested that although the mutation of *APP* and *PSEN1* in neurons enhanced the vulnerability of isoflurane-induced caspase-3 activation in mice neurons, the mutation did not increase the vulnerability of the isoflurane-induced caspase-3 activation and elevation of cytosol cytochrome c levels in mice NPCs.

Isoflurane may induce caspase-3 activation via up-stream mechanisms other than mitochondria, which can also be assessed in our established model/system. Using human fetal cortical neural progenitor cells, Peng et al. have found that tumor necrosis factor-related apoptosis-inducing ligand receptor 2 is highly expressed on human NPCs derived from fetal cortex. The inhibitors of apoptosis proteins are also highly expressed in the human NPCs. Finally, cellular inhibitors of apoptosis protein may protect NPCs against apoptosis and caspase-3 activation (Peng et al., 2005). These findings suggest that isoflurane may also induce caspase-3 activation by affecting the tumor necrosis factor-related apoptosis-inducing ligand receptor 2 and/or the cellular inhibitors of apoptosis protein. Future studies to test the hypothesis in our established model/system of mice NPCs and neurons are warranted.

There are several limitations in the current studies. First, we did not assess the levels of total cytochrome c or other internal controls in the studies, and we did not compare the effects of isoflurane on other mitochondrial functions, e.g., levels of reactive oxygen species, opening of mitochondrial permeability transition pore, mitochondrial membrane potential, between mice NPCs and neurons. However, the objective of the current studies was to establish a model/system. The established model/system will allow us to systematically determine and compare the effects of isoflurane on cellular function between NPCs and neurons, which will likely facilitates the elucidation of the up-stream mechanism by which isoflurane induces caspase-3 activation. Second, we did not compare the change of the amount of cytochrome c levels inside the mitochondria. This is because our previous studies have illustrated that there could be minimal changes in the mitochondrial cytochrome c level following isoflurane treatment in H4 human neuroglioma cells (Zhang et al., 2010). Third, we did not investigate the effects of different concentrations of isoflurane on caspase-3 activation in the studies. This is mainly because our previous studies have shown that treatment with 2%, but not 1%, isoflurane for 6, but not 3 h is able to induce caspase-3 activation in cultured cells and neurons (Xie et al., 2006a,b; Zhen et al., 2009). Finally, there is currently no satisfactory way to extrapolate caspase-3 findings in cultured neurons and NPCs to the *in vivo* brain. The *in vitro* findings in the established model/system in the neurons and NPCs will require further studies to determine the *in vivo* relevance.

In conclusion, we have found that the treatment with 2% isoflurane for 6 h can induce caspase-3 activation in mice neurons but not in mice NPCs. These data have established a model/system to determine the up-stream mechanism by which isoflurane induces caspase-3 activation. Moreover, we have found that the isoflurane treatment increases the cytosol cytochrome c levels in mice neurons but not in mice NPCs. These results have demonstrated that we are able to use the established model/system to investigate the underlying mechanisms of isoflurane-induced caspase-3 activation. These findings will hopefully promote more studies to investigate anesthesia neurotoxicity.

## Author contributions

Zhongcong Xie, Deborah J. Culley, Gregory Crosby, and Yiying Zhang conceived and designed the project. Yiying Zhang, Chuxiong Pan, Xu Wu, and Yuanlin Dong performed all the experiments and prepared the figures. Zhongcong Xie wrote the manuscript. All authors reviewed the manuscript.

## Conflict of interest statement

The authors declare that the research was conducted in the absence of any commercial or financial relationships that could be construed as a potential conflict of interest.
